# Assessment and Treatment of the Anorexia of Aging: A Systematic Review

**DOI:** 10.3390/nu11010144

**Published:** 2019-01-11

**Authors:** Natalie J. Cox, Kinda Ibrahim, Avan A. Sayer, Sian M. Robinson, Helen C. Roberts

**Affiliations:** 1 Academic Geriatric Medicine, Faculty of Medicine, University of Southampton, Southampton SO16 6YD, UK; avan.sayer@newcastle.ac.uk (A.A.S.); hcr@soton.ac.uk (H.C.R.); 2 NIHR Southampton Biomedical Research Centre, University of Southampton and University Hospital Southampton NHS Foundation Trust, Southampton SO16 6YD, UK; 3 NIHR Collaboration for Leadership in Applied Health Research and Care (NIHR CLAHRC) Wessex, University of Southampton, Southampton SO16 7NP, UK; k.ibrahim@soton.ac.uk; 4 AGE Research Group, Institute of Neuroscience, Newcastle University, Newcastle upon Tyne NE4 5PL, UK; 5 NIHR Newcastle Biomedical Research Centre, Newcastle upon Tyne Hospitals NHS Foundation Trust and Newcastle University, Newcastle upon Tyne NE4 5PL, UK; 6 MRC Lifecourse Epidemiology Unit, University of Southampton, Southampton SO16 6YD, UK; smr@mrc.soton.ac.uk

**Keywords:** appetite, anorexia, treatment, assessment, older people, frailty, nutrition, systematic review

## Abstract

(1) Background: Appetite loss in older people, the ‘Anorexia of Aging’ (AA), is common, associated with under-nutrition, sarcopenia, and frailty and yet receives little attention. This review had two aims: describe interventions for AA and their effectiveness, and identify the methods of appetite assessment. (2) Methods: Study inclusion: participants aged ≥65, intervention for AA, and appetite assessment, any design, and comparator. Exclusion: studies on specific health cohorts. Searches in four databases with hand searching of references and citing works. Two researchers independently assessed eligibility and quality. (3) Results: Authors screened 8729 titles, 46 full texts. Eighteen articles were included describing nine intervention types: education (*n* = 1), exercise (*n* = 1), flavor enhancement (*n* = 2), increased meal variety (*n* = 1), mealtime assistance (*n* = 1), fortified food (*n* = 1), oral nutritional supplement (ONS) (*n* = 8), amino acids (*n* = 1), and medication (*n* = 2). Three studies evaluated combinations: education + exercise, ONS + exercise, and ONS + medication. Five intervention types exhibited favorable effects on appetite but in single datasets or not replicated. Appetite was assessed predominantly by Likert (*n* = 9), or visual analogue scales (*n* = 7). (4) Conclusions: A variety of interventions and methods of appetite assessments were used. There was a lack of clarity about whether AA or undernutrition was the intervention target. AA is important for future research but needs standardized assessment so that effectiveness of a range of interventions can be fully explored.

## 1. Introduction

The loss of appetite experienced by older people has been largely attributed to the aging process and is often termed the ‘anorexia of aging’ (AA) [1]. The reported prevalence of AA ranges from up to 25% in home dwellers to 62% in hospital populations and 85% in nursing home populations [2].

**Figure 1:**
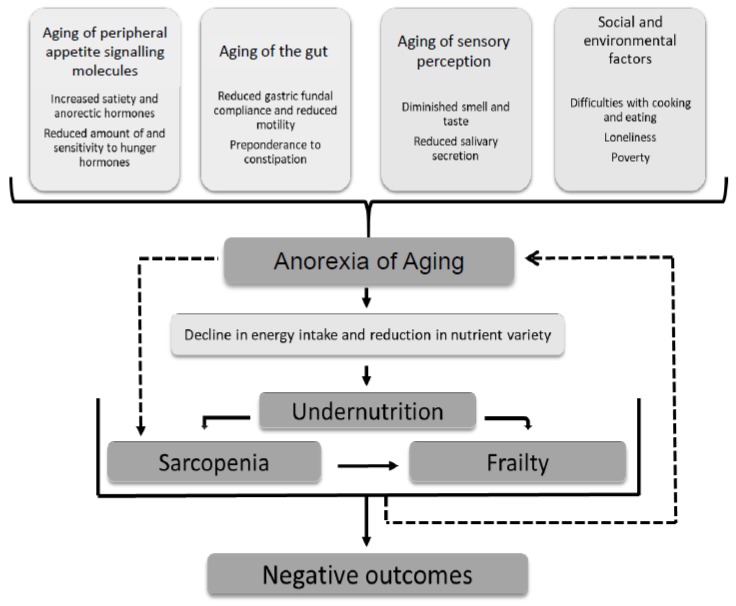
Multi-factorial causes of the anorexia of aging. Consequences are mostly attributable to subsequent undernutrition but there is some evidence for an independent association with sarcopenia [2,8,9,10,11,12,13,14,15,16].

AA has been linked to a number of important sequelae, predominantly due to poor oral intake and reduction in a variety of nutrients including protein, fiber, whole grains, fruits, and vegetables [3,4]. The consequences of AA include the development of subsequent undernutrition, immunosuppression, sarcopenia, and frailty (which can reciprocally worsen appetite further). This ultimately leads to adverse outcomes with higher rates of morbidity and mortality [3,5,6,7,8]. The causes of AA include changes in peripheral hormone signaling, gut motility, and sensory perception due to aging as well as social and environmental factors (Figure 1) [2,8,9,10,11,12,13,14,15,16].

The terms undernutrition and AA are often used interchangeably, rather than the former being recognized because of the other. Currently, clinical efforts are concentrated more on identifying patients at risk of undernutrition, with AA receiving little attention. Undernutrition screening tools such as the Mini-Nutritional Assessment Short Form (MNA-SF) and Malnutrition Universal Screening Tool (MUST) are based on body mass index as well as weight loss and dietary intake, which are parameters that are also often thought of as a marker of appetite [17,18,19,20,21,22]. This lack of distinction between AA and undernutrition has led to research overlap with studies looking to address AA but then defining participants and intervention targets in terms of weight loss and oral intake rather than appetite assessment [18,22]. However, the amount a person consumes may also be subject to other factors such as masticatory, functional ability, and environmental factors rather than appetite alone, and so can be misleading [18].

To enable accurate identification of AA, appetite needs to be assessed. However, this is not routinely done in clinical practice. Different appetite assessments have been devised for research in both over-nutrition and under-nutrition [7,23,24,25,26]. Currently, it is unclear which method is best to use when evaluating clinical outcomes and treatment efficacy for the older population. Accurate assessment of AA and a focus on interventions, particularly prior to significant weight loss, could potentially be an approach to prevent the onset of undernutrition and slow the frailty trajectory [26].

This systematic review had two aims. The first aim was to describe current interventions for AA and their reported effectiveness in the older population, and the second aim was to identify the methods of the appetite assessment used.

## 2. Materials and Methods

This systematic review was carried out using the methods recommended by the Preferred Reporting Items for Systematic reviews and Meta-Analyses (PRISMA) statement [27]. The review was registered on the international prospective register of systematic reviews (PROSPERO) ID number: CRD42018096302. The full protocol is available upon request.

### 2.1. Study Inclusion

**Figure 2:**
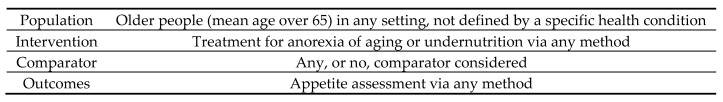
PICO statement for study inclusion.

The criteria for inclusion is presented in Figure 2. Articles relating to studies with any design or setting were included if they measured appetite (even if it was not the primary outcome), in people with a mean age of 65+ years and described a treatment for AA. Due to the amount of interchangeability between the terminology of undernutrition and AA, studies reporting an intervention for undernutrition (also termed malnutrition) that assessed the appetite were included. Articles in any language were considered.

Studies focusing on a cohort with a specific physical or mental health condition known to impact on appetite (including cancer, chronic obstructive pulmonary disease (COPD), heart failure, renal failure, depression, anorexia nervosa, and dementia) were excluded. Studies focusing on understanding the physiological mechanisms of AA were also excluded.

### 2.2. Data Collection

A search was run in four online databases: EMBASE and MEDLINE via the OVID SP platform, the Web of Science and CINAHL via the EBSCO platform. The searches were run from database conception until 11 May, 2018 with no limits on the publication type. An example search strategy for the MEDLINE search is included (Appendix A).

Screening of titles and then abstracts for relevance was performed independently by two authors (N.J.C. and K.I.) using the Rayyan electronic platform [28]. Following each stage, there were conferring and disputed texts that were taken forward to the next stage. Full texts of potentially relevant abstracts were reviewed against inclusion and exclusion criteria. Hand searching of the reference list and citing works of included texts and relevant reviews was completed. All articles arising from a single dataset were evaluated and the most comprehensive article related to appetite assessment was included.

### 2.3. Data Analysis

Data from included studies were abstracted into a pre-defined template (designed by N.J.C.). Authors were contacted to obtain further information. Publication quality was assessed using the standardized Joanna Briggs Institute checklists for each study type giving a total score of 13 for randomized controlled trials (RCTs), nine for quasi-experimental studies, and 10 for qualitative studies [29].

## 3. Results

**Figure 3:**
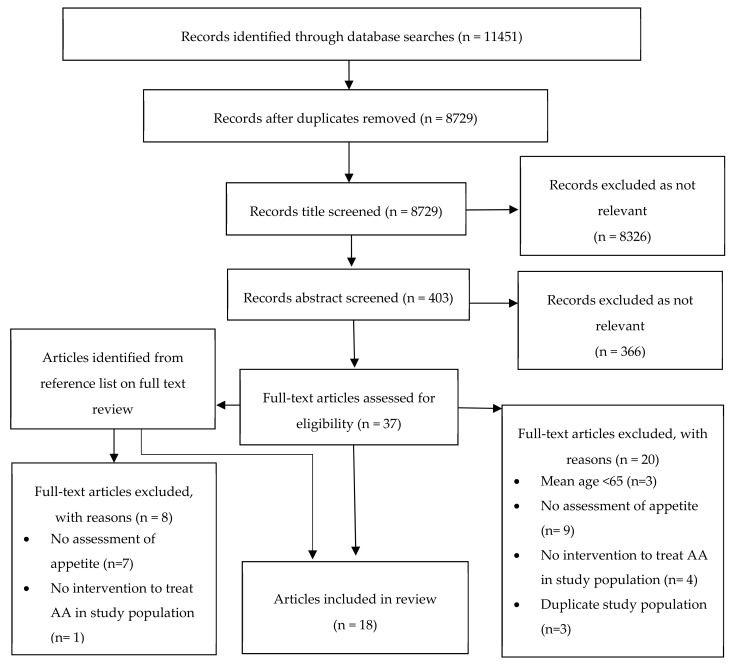
Flow diagram for screening and eligibility of studies for inclusion.

The initial search identified 8729 articles following removal of duplicates. All titles were screened for relevance and 403 abstracts were reviewed. Forty-six full text articles were reviewed with 18 studies meeting inclusion criteria [30,31,32,33,34,35,36,37,38,39,40,41,42,43,44,45,46,47]. A summary of screening and eligibility is included in Figure 3.

The reporting format consisted of 17 journal articles and one conference abstract [36]. Quality scores ranged from 5/13 to 13/13 for RCTs (the conference abstract scored poorly due to insufficient data), 9/9 for all within subject designs, and 8/10 for the qualitative study (Appendix B). No studies were excluded following quality assessment [30,31,32,33,34,35,36,37,38,39,40,41,42,43,44,45,46,47].

From the 18 included studies, nine different types of intervention for AA were identified. These were grouped into categories devised by the authors, judged on clinical relevance and similarity. The authors accept that there are alternative ways in which the interventions could be grouped but this method enabled synthesis with a clinical focus (Table 1). Due to the heterogeneity of study methodology and results, a meta-analysis was unachievable. Results are presented as a narrative synthesis of study characteristics including treatment strategies for AA with a reported effect on appetite and then methods were used to assess appetite. A summary of the included studies, grouped by intervention category, is provided (Table 2).

### 3.1. Study Characteristics

The studies were comprised of 12 randomized controlled trials, five within subject design studies (participants receiving both intervention and control), and one qualitative study [30,31,32,33,34,35,36,37,38,39,40,41,42,43,44,45,46,47]. A number of countries were represented including the United States, United Kingdom, France, The Netherlands, Sweden, Norway, Australia, and Japan. The sample populations were from our own home (*n* = 6), care home (*n* = 6), acute hospital (*n* = 5), and rehabilitation (*n* = 3) (some studies drew their sample from multiple settings. See Table 2) [30,31,32,33,34,35,36,37,38,39,40,41,42,43,44,45,46,47]. Two studies followed participants across settings: one from rehabilitation to their own home [30], and one from a hospital stay and into the community [33].

The studies totaled 1115 participants with individual sample sizes ranging from 12 to 185 and a mean age range of 74–87 years [30,31,32,33,34,35,36,37,38,39,40,41,42,43,44,45,46,47]. Sixteen studies reported the mean body mass index (BMI) of participants, which ranged from 19.3 to 30 but was mostly below 25 [30,31,32,33,34,35,36,37,38,39,40,41,43,44,46,47]. Seventeen of the study samples were defined as being either undernourished (using nutritional assessment tools, BMI, or weight loss) or having a self-reported poor appetite at baseline [30,31,32,33,34,35,36,37,38,39,40,41,42,43,44,45,47]. Only four studies [31,32,41,44] reported eating difficulty or artificial feeding as exclusion criteria. Samples were predominantly female with only three studies recruiting mostly men [37,43,45]. Two studies did not report the sex of participants [32,36].

### 3.2. Treatment Strategies for Anorexia of Aging and Reported Effect on Appetite

Included studies reported on a range of interventions for AA. The nine different types of intervention have been broadly categorized by the authors into education, exercise, meal adjustments, supplementation, and medications (Table 1). Some of the interventions were used in combination. When reported individually for a study arm, effects on appetite are described individually. Otherwise, they are described for the combination (Table 1).

#### 3.2.1. Education (*n* = 1)

Andersson et al. assessed a three-month education program [30]. This entailed an individual nutritional plan for dietary requirements and intake pre-discharge from the hospital and then post-discharge counseling [30]. Counseling addressed the eating environment, motivation, and support to increase intake, food preparation, food choices, and also undefined social and psychological factors [30]. Thirteen percent of participants were lost to follow up and comply with the intervention, which was assessed but not stated. There was no significant effect on appetite when compared to a control group receiving the usual care (*p* > 0.05) [30].

#### 3.2.2. Exercise (*n* = 1)

The first arm of de Jong et al. [34] considered a 17-week community based exercise program. This required 45-min group sessions twice a week focusing on muscle strength, coordination, flexibility, and endurance [34]. Compliance and physical activity levels during the intervention were not assessed. The authors reported no effect on appetite compared to controls attending a class with creative activities (*p* = 0.61) [34].

#### 3.2.3. Meal Adjustments (*n* = 4)

Four studies adjusted the participant’s meal or mealtime [39,42,44,46]. Mathey et al. [39] used flavor enhancement with four different flavors (chicken, beef bouillon, turkey, and lemon butter for fish) depending on the meal constituents, all of which contained monosodium glutamate. This was sprinkled over the entire main meal for 17 weeks in a care home. The authors reported increased daily feelings of hunger among participants, compared to controls having the usual meal and with the participant’s baseline appetite (*p* < 0.05) [39]. Increasing the flavor of meals was also studied by Best et al. on a community dwelling of older people [46], who used seasoning on one occasion (two spoonfuls of a choice of branded recipe) or sauce on another (100 g of a choice of branded recipe) on a meal of chicken, vegetables, and mashed potato [46]. In comparison to a control meal of the same ingredients, there was no difference in pre or post meal appetite domains of ‘hunger’ or ‘desire to eat’ (hunger *p* = 0.28, *p* = 0.65, desire to eat *p* = 0.36, *p* = 0.15) [46]. Wijnhoven et al. [44] changed the constituents of single meals to increase their variety for community dwellers. Meals with greater variety had three different selections of vegetables of three different colors, three different types of meat (or fish), and three different types of starch all-together on one plate. They reported no change in appetite compared with a control meal of only one variety of vegetable, meat, and starch (P not reported) [44].

Robison et al.’s qualitative study [42] explored the effect of one year of trained mealtime volunteer assistants helping hospital patients to eat on their appetite and food intake. The impact of mealtime assistants on appetite was not reported, but themes of poor appetite were prevalent in interviews from both the pre and post intervention samples with no apparent improvement following volunteer help [42].

#### 3.2.4. Supplementation (*n* = 10)

Supplementation included oral nutritional supplements (ONS), an amino acid-precursor, and fortified food. ONS were assessed in eight studies (an individual arm of two) [31,33,34,35,36,37,43,47]. All ONS differed in constituents when specified and included fatty emulsion, high protein, low protein, high fat, high carbohydrate, and micronutrient dense formulas [31,33,34,35,36,37,43,47]. The total 24-h energy content ranged from 200‒419 Kcal in 250 mL or 30 mL portions. None of the ONS were directly comparable in terms of constituent ingredients, energy content, and volume [31,33,34,35,36,37,43,47]. ONS showed mixed effects on appetite. No effect was seen for ONS with unspecified constituents (*p* values not reported) or high in micronutrients (*p* = 0.17) when compared to controls receiving usual care or standardized dietary advice sheets [31,34,36]. In Carlsson et al.’s study, the arm investigating individuals’ effects of a high protein ONS [33] reported no effect on appetite compared with controls receiving the usual care at six months (*p* value not reported) [33]. An increase in the appetite domains ‘desire to eat’ and ‘hunger’ were reported in two different studies using fat emulsion ONS, compared to controls receiving usual care (desire to eat *p* = 0.021 [35], ‘hunger’ *p* = 0.026. [47]). A transient decrease in the domain of ‘hunger’ following a high fat and a high protein ONS after 4.5 h when compared to participant’s baseline was reported by Ryan et al. (*p* = 0.04) [43] and Irvine et al. (*p* = 0.01) [37], respectively. This did not persist in measurements at later time points [37,43]. Three of the studies using ONS reported a loss to follow up of participants due to product intolerance (numbers not specified) [35,37,43].

Brocker et al. evaluated the amino acid pre-cursor, ornithin oxoglutarate [32]. Home dwellers following recent hospital discharge were given 10 g once a day with controls receiving placebo [32]. An increase in the appetite domains ‘overall appetite’ and ‘appetite for meat’ was observed when compared to controls at 60 days (*p* < 0.001, *p* < 0.001) [32]. Pouyssegur et al. [40] assessed fortified food for care home residents, in the form of cookies between meals delivering 244 Kcal energy in 24 h. The authors reported an increase in the ‘appetite’ domain after 18 weeks when compared to participant’s baseline (*p* = 0.009) [40].

#### 3.2.5. Medications (*n* = 2)

The effect on appetite of the progestogen medication megestrol acetate (MA) was evaluated in two double blind placebo RCTs including one on care home residents and another on community dwellers following recent hospital discharge [41,45]. MA was prescribed in either 200, 400, or 800 mg doses over 24 h with mixed results. At doses of 800 mg, when compared to controls, Yeh et al. described an increase in appetite at 12 weeks (*p* = 0.004) [45] while Reuben et al. observed no difference when compared to controls at three or six weeks for the range of doses from 200 to 800 mg (*p* = 0.07) [41]. Reuben et al. did, however, report an increase from the participant’s baseline in the domains of ‘appetite’ and ‘appetite at start of last meal’ with the 800 mg and 400 mg doses, respectively (*p* = 0.04, *p* = 0.02) [41]. The authors discussed adverse outcomes. Reuben et al. reported significantly lower cortisol levels compared to controls at 20 days (400 mg *p* = 0.003, 800 mg *p* = 0.02) but no clinical symptoms of adrenal insufficiency [41]. They also observed venous thromboembolism (*n* = 2) and diarrhea (*n* = 3) [41]. Yeh et al. reported no statistically significant differences compared to controls for events related to drug therapy, and no significant difference on mortality (*p* values not reported) [45].

#### 3.2.6. Combined Interventions (*n* = 3)

Kimura et al. [38] evaluated the combination of exercise and education for home-owning dwellers over a 14-month period. This comprised of one-hour exercise classes every two weeks focusing on muscle strengthening, with at-home exercise recommendations and daily recording with feedback. Alongside were five 30-min lectures on dietary habits with participants recording daily intake, which was reviewed with motivational comments [38]. The authors found no change in appetite in the intervention group, compared to controls receiving usual care (*p* = 1.0) [38].

The third arm of the de Jong et al. study evaluated 17 weeks of micronutrient dense ONS in combination with the described exercise program [34]. There was no change in appetite compared to controls receiving regular products and classes with creative activities (*p* value not reported) [34].

The effect of a protein rich ONS (244 Kcal in 24 h) in combination with the anabolic steroid nandrolone decanoate (25 mg every three weeks) was assessed in a second arm of Carlsson et al. [33]. They observed no difference in appetite compared to controls receiving usual care (*p* not reported) but an increase from baseline appetite assessment for six of the 15 participants having the combination (*p* = 0.02) [33]. Adverse incidents relating to nandrolone decanoate were not reported in the article [33]. An article on the same study cohort noted a transient rise of serum calcium, which reverted without therapy change, and incidence of urinary tract infections. A diagnosis was also present in the control group with no measure of difference between the two [48].

### 3.3. Methods of Appetite Assessment

A range of methods were used to assess appetite among studies that could be categorized into Likert scales, visual analogue scales (VAS), and a qualitative method. All of the studies assessed the participant’s appetite at recruitment. Subsequent assessment then ranged from immediately post intervention (e.g., immediately post meal consumption) to 14 months for the longest intervention. There was no association between settings, timing of measurement or type of intervention, and the appetite assessment used [30,31,32,33,34,35,36,37,38,39,40,41,42,43,44,45].

#### 3.3.1. Likert Scales

Nine studies used the Likert scale method to assess appetite. Andersson et al. [30] used the Disease Related Appetite Questionnaire (DRAQ). This questionnaire, based on the Council on Nutrition Appetite Questionnaire (CNAQ), was created for COPD patients [26,30,49]. The DRAQ contains 10 domains each using a five point Likert scale. Domains included semi-quantification of appetite, day-to-day variations in appetite, food tastes, frequency of eating, presence of nausea, and impact of mood or co-existing disease on food intake with a maximum score of 50 corresponding to a good appetite.

Mathey et al. [39] and de Jong et al. [34] both assessed appetite with the Appetite, Hunger, and Sensory Perception Questionnaire (AHSPQ). This 29 domain questionnaire, using a five-point Likert scale, correlates with weight change in a community dwelling among older people [50], and in the de Jong study correlated with self-reported energy intake by participants (*p* < 0.0002) [34]. Domains are grouped into the present taste perception, the present smell perception, the present smell perception compared with the past, appetite, and daily feelings of hunger. A maximum score of 145 corresponds to positive feelings.

A further six studies used unnamed Likert scales to assess appetite [31,33,41,44,45,46]. The scales had differing domains. These included ‘overall appetite,’ ‘appetite at the start of the last meal,’ ‘hunger,’ ‘hunger at the start of the last meal,’ ‘thirst,’ ‘fullness/satiation,’ ‘prospective consumption,’ and ‘desire to eat.’ The domain of ‘overall appetite’ was used widely but no domain was common to all six of these Likert scales. The Likert scales also varied in the number of rating points, ranging from five to nine. There were no references to validity. Two had been used previously in healthy older men [31], and a cancer cohort [45].

#### 3.3.2. Visual Analogue Scales (VAS)

Seven studies assessed appetite by VAS [32,35,36,37,40,43,47]. These numerical scales were reported in different ways as either 10-point or 100-point lengths. Multiple different domains were measured including ‘overall appetite,’ ‘hunger,’ ‘fullness,’ ‘desire to eat,’ ‘prospective consumption,’ ‘preoccupation with food,’ ‘thirst,’ ‘stress,’ and ‘cold’. The domains of ‘hunger,’ ‘fullness,’ ‘desire to eat,’ and ‘preoccupation with food’ predominated but no domain was used across all seven studies. Two studies screened participants on their ability to complete a VAS before inclusion but did not report how many were excluded on this basis [37,43]. Three studies referenced the ability of their VAS to predict oral intake in healthy young people [23,35,43,47], and one cited oral intake in a cancer cohort [40,51].

#### 3.3.3. Qualitative Approach

Robison et al. [42] assessed subjective appetite perceptions by semi-structured interviews. The interviews broadly covered the topics of appetite, choosing what to eat, managing at mealtimes and food, and fluid intake during the hospital stay [42]. The authors used prompts to explore interviewee’s experiences pre and post intervention. Framework thematic analysis then captured the range of perspectives [42].

#### 3.3.4. Undefined Method

Kimura et al. [38] assessed appetite using an undefined method, which reported the outcome in a ‘yes/no’ nominal style and made no reference to the tool’s validity.

## 4. Discussion

This review identified 18 studies with an intervention for AA and appetite assessment enabling evaluation of nine different intervention types to improve appetite [30,31,32,33,34,35,36,37,38,39,40,41,42,43,44,45,46,47]. These studies were carried out in different countries with participants from different settings including hospital, rehabilitation, care homes, and own home. The mean ages of participants were over 74 years with a BMI predominantly below 25. The studies displayed heterogeneity in methodology, assessment, and intervention type so pooling of the data for meta-analysis was not possible.

The nine different types of intervention for AA were broadly categorized into education (nutritional counselling), exercise (exercise programs), meal adjustments (flavor enhancement, increased variety, mealtime assistance), supplementation (ONS, amino acid precursor, fortified food), medication (megestrol acetate or nandrolone decanoate medication) and combinations. Of the nine different types of intervention, five exhibited some favorable effects on appetite (flavor enhancement, ONS, an amino acid precursor, fortified food, and megestrol acetate medication) when compared to controls or from baseline. However, findings were either in single datasets or not replicated across studies.

Appetite was assessed in a number of different ways, predominantly using Likert or visual analogue scale methods but with a range of different domains and scoring systems. Some of the methods used have been validated in older people, but generally methods were created for other populations.

This review has again highlighted that many studies combine the concepts of AA and undernutrition. There was variation in the authors’ treatment intentions, whether AA or undernutrition, when measuring appetite as an outcome. Appetite and outcome of oral intake when measured were also often discussed together even though, largely, studies did not control for other factors that may impact on eating such as masticatory ability or dysphagia. The terms undernutrition and AA were often used interchangeably rather than undernutrition being recognized due to AA. Lack of clarity on this is important since some people with AA may not yet be undernourished but rather be at risk of developing it (as seen with Mathey et al.’s study sample, with a poor appetite, but a BMI > 25) and so could be missed if undernutrition is the sole focus [26,39]. A focus on AA with treatment of this specific group may reduce the onset of weight loss. However, currently there is a gap in the literature on this sub-group, due to participants often being identified for study inclusion by poor oral intake or weight loss. Observational data on the covariates of those with a poor appetite who are not undernourished would be valuable, to fully understand this cohort and enable targeted interventions.

Among interventions for AA, the two studies addressing education and exercise did not suggest any effect on appetite. However, the studies had significant limitations related to a lack of reporting of compliance measures and levels of physical activity achieved for the exercise programs. Adjustments to participant’s meals gave mixed results, but the introduction of flavor enhancers may have a beneficial effect counteracting diminished sensory function attributable to aging to increase food palatability [39,52].

Studies assessing ONS were the largest sub-group, consistent with current clinical guidance on the management of undernutrition. The effects on appetite were mixed, but there seems to be some consensus that additional ONS only transiently reduces appetite, if at all [31,35,36,37,43]. This effect on appetite is also reported in controlled studies assessing the physiology of AA, which reinforces the view that addition of ONS increases overall energy intake [53,54,55]. However, losses to follow-up due to product aversion occurred in ONS studies (numbers not reported) [35,37,43]. This finding is in line with opinion that ONS can be poorly tolerated [56]. The amino acid precursor and fortified food studies both reported an increase in appetite [32,40]. Patients who were compared to ONS better tolerate fortified food [57] and a potential associated increase in appetite suggests it warrants further exploration.

The effect of medications was also considered in this review with evidence that megestrol acetate (MA) had a stimulatory effect on appetite [41,45]. However, MA has been associated with safety concerns in older people including adrenal suppression, venous thromboembolism, hyper- and hypoglycaemia, changes in mental state, diarrhea, insomnia, and osteoporosis [41,58,59,60]. The side effect profile of anabolic agents such as nandrolone decanoate, is also considerable, including fluid retention, liver injury, and prostatic hypertrophy [59]. These findings caution against the use of these medications for AA.

The lack of reproducibility for interventions trialed in more than one study may, in part, be due to the need for multi-component interventions for AA, in view of the number and diversity causes (Figure 1). Only three of the included studies combined interventions, all of which had just two components and for the lifestyle interventions included limitations, which impacted on the assessment of compliance and utility. There is currently a gap in the literature on multi-component interventions, which aim to address different causes of appetite loss. This would be beneficial and may yield positive results.

Appetite was assessed in different ways. Of the first main group, the Likert scales, the Appetite, Hunger and Sensory Perception (AHSPQ) questionnaire has validity among community dwelling older people correlating with weight change and self-reported intake [34,50]. However, it has subsequently been shown to be difficult to adapt to undernourished older populations in different settings [61]. Thus, the Council on Nutrition Appetite Questionnaire (CNAQ) with its reliable shortened derivative, the Simplified Nutritional Appetite Questionnaire (SNAQ) were developed from the AHSPQ and have been shown to predict weight loss in community dwelling older people [26]. The SNAQ consists of four domains: appetite, fullness, taste of food, and meal frequency. A score of ≤14 indicates a risk of 5% weight loss in six months [26]. It has validity against the nutritional assessment tools Subjective Global Assessment (SGA) and Mini-Nutritional Assessment (MNA) but may ‘over predict’ undernutrition [62,63]. This perhaps reflects the nature of the SNAQ as more of an appetite assessment tool where a proportion of those with AA may not yet have experienced significant weight change, but who, nevertheless, may proceed onto it. The SNAQ originated in a community setting but has shown reliability, validity, and ability to predict inadequate intake and higher risk of morbidity and mortality in the hospital setting [5,7,26,64]. The DRAQ [30] is based on the CNAQ but adds disease-related questions since it was created for COPD patients, which makes it more relevant for measuring appetite loss due to chronic disease rather than AA.

Visual analogue scales (VAS) were the other commonly used method of appetite assessment. The VAS domains that show ability to predict eating behavior in older people are hunger, prospective consumption, and fullness [24,25]. VAS have originated from the laboratory environment assessing the physiological mechanisms of appetite control [13,24]. Studies included in this review, have also shown VAS to be transferrable to the clinical environment [35,37,40,43]. However, screening of ability to complete a VAS in two of the included studies suggested a potential limitation of its feasibility of use for older people [37,43]. This has also been seen when VAS have been used for the assessment of pain in older populations, and a high number of errors in completion have been reported [65]. These findings suggest that VAS may not be ideal in a clinical setting where staff need a quick and easy tool.

This review has focused on appetite assessment methods for older people in the context of an intervention. When looking at non-intervention studies, there is a large overlap in methods. Most commonly used are semi-structured interviews [66,67,68,69], the CNAQ or SNAQ (including different language derivatives) [5,7,70,71,72,73,74,75,76,77], and Likert scales with differing domains and rating points [3,20,78,79,80,81,82,83,84]. Appetite can also be assessed as a part of some nutritional assessment tools such as the Short Nutritional Assessment Questionnaire 65+ (SNAQ 65+) [83] or MNA. However, this can also add to the lack of clarity between AA and undernutrition.

### Strengths and Limitations of the Review

This systematic review was carried out following the recommendations of the PRISMA statement [27]. Two researchers worked independently to assess studies for inclusion and quality. Hand searching was undertaken to ensure appropriate acquisition of studies and no limits on publishing language were set. The authors of two included studies were also contacted with one response and further information. However, the search did not include the grey literature where sources such as service improvement projects may have been identified.

The included articles were of variable quality although the studies were representative of the older population across settings. However, the interventions were variable and some were only represented in single samples. Likewise, the appetite assessment tools used also varied and were often not validated for use in older people.

## 5. Conclusions

The review identified only 18 studies with an intervention for AA and appetite assessment. There was a lack of clarity about whether AA or undernutrition was the target of interventions. Among a variety of intervention types, flavor enhancement, ONS, an amino acid precursor, fortified food, and megestrol acetate showed a favorable effect on appetite but only in single datasets or not reproduced across studies. Currently, the side effect profiles of medications studied and the burden of polypharmacy among this population make this an unattractive option. However, flavor enhancement and supplementation particularly in the form of fortified food could be potential avenues of interest, together with a more rigorous assessment of the impact of lifestyle measures such as exercise. The review identified four different methods of appetite assessment, which were predominantly Likert and visual analogue scales but in a variety of forms. AA is an important area for future research but assessment needs to be standardized so that the effectiveness of a range of interventions can be fully explored.

## Figures and Tables

**Table 1:** Categorization of types of intervention for anorexia of aging with included studies.

Intervention Category (Number of Studies)	Intervention Type with Included Studies
Education (*n* = 1)	Nutritional counseling
	Andersson et al. [30]
Exercise (*n* = 1)	Exercise program
	De Jong et al. (arm 1) [34]
Meal Adjustments (*n* = 4)	Flavor enhancement
	Best et al. [46]
	Mathey et al. [39]
	Increased meal variety
	Wijnhoven et al. [44]
	Mealtime volunteer assistance
	Robison et al. [42]
Supplementation (*n* = 10)	Oral nutritional supplement
	Boudville et al. [31]
	Carlsson et al. (arm 1) [33]
	De Jong et al. (arm 2) [34]
	Faxen-Irving et al. [35]
	Hubbard et al. [36]
	Irvine et al. [37]
	Ryan et al. [43]
	Tylner et al. [47]
	Amino acid pre-cursor
	Brocker et al. [32]
	Fortified Food
	Pouyssegur et al. [40]
Medication (*n* = 2)	Medication (megestrol acetate)
	Reuben et al. [41]
	Yeh et al. [45]
Combinations (*n* = 3)	Education & exercise
	Kimura et al. [38]
	Exercise & oral nutritional supplement
	De Jong et al. (arm 3) [34]
	Oral nutritional supplement & medication (nandrolone decanoate)
	Carlsson et al. (arm 2) [33]

**Table 2:** Summary of included studies grouped by intervention category.

Author, Reference, Year, Country	Study Design & Intervention Length	Setting & Participants	Age *	Appetite Assessment (Time Points Measured)	Intervention	Control	Effect on Appetite
Andersson et al. [30] 2017 Norway	Open RCT (3 months)	Rehabilitation + Own home *N* = 100, F ^#^ = 62% BMI * = 20.2 (3.3)	75 (8.7)	DRAQ 10 item Likert 1–5 (Recruitment, 3 months)	Education: -Nutrition plan pre-discharge and counseling post discharge	Usual care	No change (*p* > 0.05)
Best et al. [46] 2011 United Kingdom	Within subject design (3 sessions)	Own home *N* = 18, F ^#^ = 77% BMI * = 30 (U/K)	77 (U/K)	Likert scale 1–5: Hunger, desire to eat (Pre-test and post-test meal)	Meal adjustment: -Seasoning (2 spoonful’s of choice of branded recipe) -Sauce (100 g of choice of branded recipe)	Control meal: -Same basic meal constituents	No change pre or post meal Hunger (*p* = 0.28, *p* = 0.65) Desire to eat (*p* = 0.36, *p* = 0.15)
Mathey et al. [39] 2001 Netherlands	Open RCT (17 weeks)	Care home *N* = 42, F ^#^ = 76% BMI * = 28.3 (7.2)	79 (5.6)	AHSPQ 29 item Likert 1–5 (Recruitment, 17 weeks)	Meal adjustment: -Flavor enhancement with four flavors containing MSG	Usual care	Increase vs. control and from baseline in daily feelings of hunger (*p* < 0.05)
Wijnhoven et al. [44] 2015 Netherlands	Within-subject design (2 sessions)	Own home + care home *N* = 19, F ^#^ = 100% BMI * = 24.8 (4.9)	84 (8)	Likert scale 1–9: Appetite, satiation (Pre and post test meal)	Meal adjustment: -Increased variety with three different varieties of meat/fish, vegetable & starch on one plate	Control meal: -One variety of components on one plate	No change (*p* not reported)
Robison et al. [42] 2014 United Kingdom	Qualitative study (1 year)	Hospital *N* = 25, F ^#^ = 100% BMI * = U/K	U/K	Individual semi-structured interviews (Purposive sample pre-intervention, 1 year)	Meal adjustment: -Mealtime volunteer assistance during a meal	Usual care	No change (Qualitative method)
Boudville et al. [31] 2004 Australia	Within-subject design (2 sessions)	Rehabilitation *N* = 14, F ^#^ = 100% BMI * = 22.6(3.4)	79 (7.5)	Likert scale 0–5: Hunger, thirst, fullness, prospective consumption, nausea (Pre and post drink and pre and post meal)	Supplementation: ONS -250 mL liquid 90 or 30 min pre-meal (250 Kcal/24 h)	250 mL water: -pre-meal	No change (*p* not reported)
Faxen-Irving et al. [35] 2011 Sweden	Open RCT (8 days)	Hospital *N* = 51, F ^#^ = 53% BMI * = 21.3(3.7)	84(7)	VAS 10 point: hunger, fullness, desire to eat, prospective consumption, preoccupation with food (Recruitment, 8 days)	Supplementation: ONS -3 × 30 mL fat emulsion based liquid with medications (419.4 Kcal/24 h)	Usual care	Increase vs. control in desire to eat (*p* = 0.021)
Hubbard et al. [36] 2008 United Kingdom	RCT (4 weeks)	Community (Undefined) *N* = 42, F ^#^ = U/K BMI * = 20.9(3.5)	84 (7)	VAS 10 point: hunger, fullness, and desire to eat (Recruitment, 4 weeks)	Supplementation: ONS -3 × 30 mL liquid (400 Kcal/24 h)	Standardized dietary advice sheet	No change (*p* not reported)
Irvine et al. [37] 2004 France	Within-Subject design (3 days)	Hospital *N* = 12, F ^#^ = 33% BMI * = 21.3(2.4)	87 (7.8)	VAS 100 point: Hunger, fullness, desire to eat, preoccupation with food, thirst, cold. (Every 30 min (t = 0 h) to lunch (t = 4.5 h), then hourly to dinner (t = 10.5 h))	Supplementation: ONS -250 mL liquid post-breakfast, High protein (HP) or low protein (LP) content (250 Kcal/24 h)	Usual care	Decrease in hunger pre-lunch, not pre-dinner for HP (*p* = 0.01) Non-significant for LP (*p* = 0.1)
Ryan et al. [43] 2004 France	Within-Subject design (3 days)	Hospital *N* = 16, F ^#^ = 38% BMI * = 20(3)	77 (8)	VAS 100 point: Hunger, satiety, desire to eat, preoccupation with food, thirst, stress, cold. (Every 30 min (t = 0 h) to lunch (t = 4.5 h), then hourly to dinner (t = 10.5 h))	Supplementation: ONS -250 mL liquid post-breakfast, High fat (HF) or high carbohydrate (HC) content (250 Kcal/24 h)	Usual care	Decrease in hunger pre-lunch, not pre-dinner for HF (*p* = 0.04) Non-significant for HC (*p* = 0.13)
Tylner et al. [47] 2016 Sweden	Open RCT with crossover (12 weeks)	Care home *N* = 39, F ^#^ = 60% BMI * = 23(3.7)	84 (7)	VAS 10 point: hunger, fullness, desire to eat, prospective consumption, preoccupation with food (Recruitment, 6, 12 weeks)	Supplementation: ONS -3 × 30 mL fat emulsion based liquid with medications (360 Kcal/24 h)	Usual care	Increase vs. control in hunger (*p* = 0.026)
Brocker et al. [32] 1994 France	Double blind placebo RCT (4 months)	Own home *N* = 185, F ^#^ = U/K BMI * = calculated Approx 19	74 (7.4)	VAS 100 point: appetite to meat, overall appetite (Recruitment, 30 and 60 days)	Supplementation: Amino acid pre-cursor -Ornithin Oxoglutarate 10 g in morning	Placebo: -Maltodextrine (same energy content)	Increase vs. control 30 days appetite for meat (*p* = 0.001) & overall appetite (*p* = 0.001) 60 days appetite for meat (*p* < 0.001) & overall appetite (*p* < 0.001)
Pouyssegur et al. [40] 2015 France	Open RCT (6 weeks)	Care home *N* = 154, F ^#^ = 80% BMI * = 19.2(2.9)	86 (7.1)	VAS 10 point: overall appetite (Recruitment, 18 weeks)	Supplementation: Fortified food -8 cookies (244 Kcal/24 h)	Usual care	Increase from baseline (*p* = 0.009)
Reuben et al. [41] 2005 United States of America	Double blind placebo RCT (63 days)	Care home + rehabilitation (Recent hospital discharge) *N* = 45, F ^#^ = 66% BMI * = 22.6(U/K)	82 (U/K)	Likert scale 0–5 or 0–4: Appetite, appetite at start of last meal, hunger at start of last meal (Recruitment 20, 42, and 63 days)	Medication: -Megestrol Acetate, 200 or 400 or 800 mg/24/h	Placebo (undefined)	No change vs. control. (*p* = 0.07) Increase from baseline in overall appetite at 20 days (*p* = 0.04), appetite at start of least meal at 42 days (*p* = 0.02)
Yeh at al. [45] 2000 United States of America	Double blind placebo RCT (12 weeks)	Care home *N* = 51, F ^#^ = 5% Weight loss of >5%, or weight 20% below IBW	76 (1.3)	Likert scale 1–5: Overall appetite (Recruitment, 12 weeks)	Medication: -Megestrol Acetate, 800 mg/24 h	Placebo (undefined)	Increase vs. control in overall appetite (*p* = 0.004)
Kimura et al. [38] 2013 Japan	Cluster RCT With crossover (14 months)	Own home *N* = 92, F ^#^ = 80% BMI * = 24.3 (2.9)	74 (5.6)	Questionnaire “yes/no” (Recruitment, 14 months)	Combination-education + exercise: -Dietary Advice -Muscle strengthening 1 h every 2 weeks + self-directed at home	Usual care	No change (*p* = 1.0)
de Jong et al. [34] 1999 Netherlands	Open RCT (17 weeks)	Own home *N* = 165, F ^#^ = 68% BMI * = 23.6(2.7)	79 (3.6)	AHSPQ29 item Likert 1–5 (Recruitment, 17 weeks)	Combination-supplementation +/or exercise: -Micronutrient dense ONS (114 Kcal/24 h). -Muscle strength, coordination, flexibility, endurance 45 min twice a week	Regular ONS (same energy) and social program: -90 min every two weeks	No significant change for Exercise (*p* = 0.61), ONS (*p* = 0.17) or Combination (*p* not reported)
Carlsson et al. [33] 2005 Sweden	Open RCT (6 months)	In hospital + discharge out to community *N* = 45, F ^#^ = 100% BMI * = 20.4(2)	83 (5)	Likert scale 0–4: overall appetite (Recruitment, 6 months)	Combination-supplementation +/- medication: -ONS liquid protein rich (200 Kcal/24 h), -nandrolone decanoate 25 mg/3 weekly	Usual care	No change for ONS and ONS + medication versus control (*p* not reported) Increase from baseline for ONS + medication (*p* = 0.02)

* Reported as mean (Standard deviation), ^#^ Percentage of participants female; BMI = body mass index, RCT = randomized controlled trial, DRAQ = Disease Related Appetite Questionnaire, U/K = unknown, AHSPQ = Appetite, Hunger, Sensory Perception Questionnaire, MSG= monosodium glutamate, VAS = Visual analogue scale, ONS = oral nutritional supplement, IBW= ideal body weight.

## Appendix A. Search Strategy for MEDLINE on OVID SP Platform

1. exp AGED/
2. elder*.mp.
3. older.mp.
4. geriatric*.mp.
5. 1 or 2 or 3 or 4
6. exp THERAPEUTICS/
7. Disease Management/
8. treat*.mp.
9. interven*.mp.
10. manag*.mp.
11. 6 or 7 or 8 or 9 or 10
12. APPETITE/
13. Appetite Regulation/
14. ANOREXIA/
15. appetite.mp.
16. anorexia.mp.
17. unplanned weight loss.mp.
18. 12 or 13 or 14 or 15 or 16 or 17
19. 5 and 11 and 18
20. exp NEOPLASMS/
21. cancer*.mp.
22. neoplas*.mp.
23. malignan*.mp.
24. carcinoma*.mp.
25. 20 or 21 or 22 or 23 or 24
26. 19 not 25
27. Anorexia Nervosa/
28. anorexia nervosa.mp.
29. 27 or 28
30. 26 not 29
31. child*.mp.
32. 30 not 31
33. Animals/not (Animals/and Humans/)
34. 32 not 33

## Appendix B

**Table A1:** Quality assessment using Joanna Briggs Institute checklist for study design.

Author, Year of Publication, Ref	JBI Checklist Used	Score/Max Score
**Andersson et al., 2017, [30]**	RCT	11/13
**Best et al., 2011, [46]**	NRES	9/9
**Boudville et al., 2004, [31]**	NRES	9/9
**Brocker et al., 1994, [32]**	RCT	13/13
**Carlsson et al., 2005, [33]**	RCT	9/13
**de Jong et al., 1999, [34]**	RCT	9/13
**Faxen-Irving et al., 2011, [35]**	RCT	9/13
**Hubbard et al., 2008, [36]**	RCT	5/13
**Irvine et al., 2004, [37]**	NRES	9/9
**Kimura et al., 2013, [38]**	RCT	9/13
**Mathey et al., 2001, [39]**	RCT	9/13
**Pouyssegur et al., 2015, [40]**	RCT	9/13
**Reuben et al., 2005, [41]**	RCT	13/13
**Robison et al., 2014, [42]**	Qualitative	8/10
**Ryan et al., 2004, [43]**	NRES	9/9
**Tylner et al., 2016, [47]**	RCT	10/13
**Wijnhoven et al., 2015, [44]**	NRES	9/9
**Yeh et al., 2000, [45]**	RCT	13/13

RCT = randomized controlled trial. NRES = non-randomized experimental studies.
